# A Quick Turn of Foot: Rigid Foot-Ground Contact Models for Human Motion Prediction

**DOI:** 10.3389/fnbot.2019.00062

**Published:** 2019-08-07

**Authors:** Matthew Millard, Katja Mombaur

**Affiliations:** Optimization in Robotics and Biomechanics, Institute of Computer Engineering, Heidelberg University, Heidelberg, Germany

**Keywords:** foot contact, musculoskeletal model, motion prediction, optimal control, multibody dynamics

## Abstract

Computer simulation can be used to predict human walking motions as a tool of basic science, device design, and for surgical planning. One the challenges of predicting human walking is accurately synthesizing both the movements and ground forces of the stance foot. Though the foot is commonly modeled as a viscoelastic element, rigid foot-ground contact models offer some advantages: fitting is reduced to a geometric problem, and the numerical stiffness of the equations of motion is similar in both swing and stance. In this work, we evaluate two rigid-foot ground contact models: the ellipse-foot (a single-segment foot), and the double-circle foot (a two-segment foot). To evaluate the foot models we use three different comparisons to experimental data: first we compare how accurately the kinematics of the ankle frame fit those of the model when it is forced to track the measured center-of-pressure (CoP) kinematics; second, we compare how each foot affects how accuracy of a sagittal plane gait model that tracks a subjects walking motion; and third, we assess how each model affects a walking motion prediction. For the prediction problem we consider a unique cost function that includes terms related to both muscular effort and foot-ground impacts. Although the ellipse-foot is superior to the double-circle foot in terms of fit and the accuracy of the tracking OCP solution, the predictive simulation reveals that the ellipse-foot is capable of producing large force transients due to its geometry: when the ankle quickly traverses its u-shaped trajectory, the body is accelerated the body upwards, and large ground forces result. In contrast, the two-segment double-circle foot produces ground forces that are of a similar magnitude to the experimental subject because the additional forefoot segment plastically contacts the ground, arresting its motion, similar to a human foot.

## 1. Introduction

Understanding the relationships between force and movement in the musculoskeletal system is key to correcting movement pathology. Though it is possible to study muscle movement *in-vivo* (Fukunaga et al., 2001; Ishikawa et al., 2003; Maganaris, 2003; Reeves and Narici, 2003) measuring muscle force *in-vivo* is not possible without invasive surgery. Currently the only way to study the motion and forces of the human musculoskeletal system is to use mathematical models and computer methods to predict quantities that cannot easily be measured.

The mathematics of optimal control can be used to predict the movements of a model (Ackermann and van den Bogert, 2010; Schultz and Mombaur, 2010; Mordatch et al., 2013). Casting human motion prediction as an optimal control problem (OCP) requires four components: a musculoskeletal model, a cost function, problem-specific constraints, and a method to solve for the vector of state and muscle force waveforms that simultaneously satisfy the equations of motion and minimize the cost function. While the underlying mathematics of multibody dynamics and optimal control is well-developed, many tissues and structures of the body are challenging to model. Though the human body contains many mechanically complex structures, it has proven particularly difficult to formulate models of foot ground contact that are both accurate and well-suited for the prediction of walking.

Inaccuracies in the model of foot ground contact affect the rest of the body because the foot forms the only boundary between the body and the ground during typical walking. The shape that a foot model makes during walking determines how ground forces are transformed into ankle torques and vice-versa. Though impressive movement predictions have been realized without an accurate foot shape (Van den Bogert et al., 2012; Mordatch et al., 2013; Koelewijn et al., 2018), differences in foot shape ultimately affect the ankle kinematics, and CoP progression. Accurately fitting the loaded shape of a foot model to experimental data is challenging because the optical markers placed on the skin of the foot move on the order of a centimeter with respect to the underlying bones (Fuller et al., 1997). Both the fitting and the simulation of viscoelastic foot models is made difficult by the widely varying stiffness of human foot pads (Aerts et al., 1995) which are compliant at initial contact (~20 N/mm) and rapidly stiffen with load (1,445 N/mm at 1 body weight). Although the literature contains some excellent examples of fitted viscoelastic foot models (Halloran et al., 2010; Pàmies-Vilà et al., 2014; Shourijeh and McPhee, 2014, 2015; Millard and Kecskeméthy, 2015; Jackson et al., 2016; Brown and McPhee, 2018), rigid foot-ground contact models are an attractive alternative: the fitting process is strictly dependent on geometry, and the numerical stiffness of the model does not change appreciably from swing to stance. A reduction in the numerical stiffness of the model is attractive because this makes the resulting optimization problem less sensitive and therefore easier to solve.

Although rigid-foot ground contact models are common in the passive dynamic walking literature (McGeer, 1990; Collins and Ruina, 2005) few rigid foot-ground models exist in the musculoskeletal modeling literature. Hansen et al. (2004) and Srinivasan et al. (2008) modeled the lower leg and foot in two-dimensions (2D) as a single rigid body that rolls on the ground using a rigid cylinder-plane contact pair. While this approach can accurately replicate the motion of the entire lower stance leg, for many applications it is not acceptable to fix the ankle joint. Although the foot has been modeled using point contacts for sprinting motions (Kleesattel and Mombaur, 2018), point contacts do not capture the rolling motion of the foot during walking (Garćıa-Vallejo et al., 2016). The foot has been modeled as a single convex cam (Ren et al., 2010; Römer, 2018; Römer et al., 2018) which contacts the ground at a single point and rolls-without-slipping across the ground plane. Ren et al. (2010)'s planar foot model closely matched the ankle position of the 12 subjects they tested (≈1 cm on average), with the largest errors appearing during heel strike (≈2.5 cm) and toe-off (≈1.5 cm). It is worth noting that a certain amount of kinematic error is expected during heel-contact and toe-off since a rigid foot ground contact model does not capture the compression of heel (Gefen et al., 2001) and forefoot pads (Cavanagh, 1999). Felis and Mombaur (2016) developed a 3D rigid foot-ground contact model using a sphere and a planar triangle to represent the heel and forefoot, respectively, but did not fit the model to experimental data. Though there are good examples of rigid foot ground contact models that interact through the ground using a single curved shape (Ren et al., 2010; Römer, 2018; Römer et al., 2018), there are no examples of fitted rigid contact models that treat the hind and forefoot separately.

Unfortunately a foot model that fits kinematic data isolation does not necessarily translate into a walking prediction that produces human-like foot ground forces. Optimal walking solutions in the literature typically have ground force profiles that deviate from experimental data, sometimes dramatically, at heel contact (Ackermann and van den Bogert, 2010; Geyer and Herr, 2010; Dorn et al., 2015) where differences between two and three times body weight are typical. Large simulated heel contact forces often arise from pairing a musculoskeletal model with a viscoelastic foot and using a problem formulation that inherently does not adjust its walking pattern in response to large contact forces. Another common problem, in which an obvious solution is not clear, are ground forces that have an appropriate magnitude but a shape that differs from experimental data (Anderson and Pandy, 2001; Ren et al., 2007; Geyer and Herr, 2010; Sreenivasa et al., 2017). To make an improvement in prediction accuracy it is necessary to distinguish error that is caused by the model of the foot from error caused by other sources.

In this work we model and evaluate two planar foot-ground contact models using three different methods in an effort to identify differences with experimental data that are caused by the foot model. The first of the rigid foot-ground contact models we consider is similar to existing works in the literature because it interacts with the ground through a single curved segment (Ren et al., 2010; Römer, 2018; Römer et al., 2018). In contrast, the second foot-ground contact model has separate contact shapes for the heel and forefoot. As is typical, we evaluate how well the kinematics of each foot model track the stance kinematics of a subject's foot in isolation. In addition, we consider how well each foot model performs as part of a whole body gait model: first, when the gait model is used as part of an optimal-control problem (OCP) to track experimental data; and second, in an OCP that predicts motion. The musculoskeletal model and foot models are described in section 2 while the detailed evaluation procedure used to assess the foot models is described in section 3. The results of the work appear in section 4 and a discussion of the results in section 5.

## 2. Model

**Figure 1:**
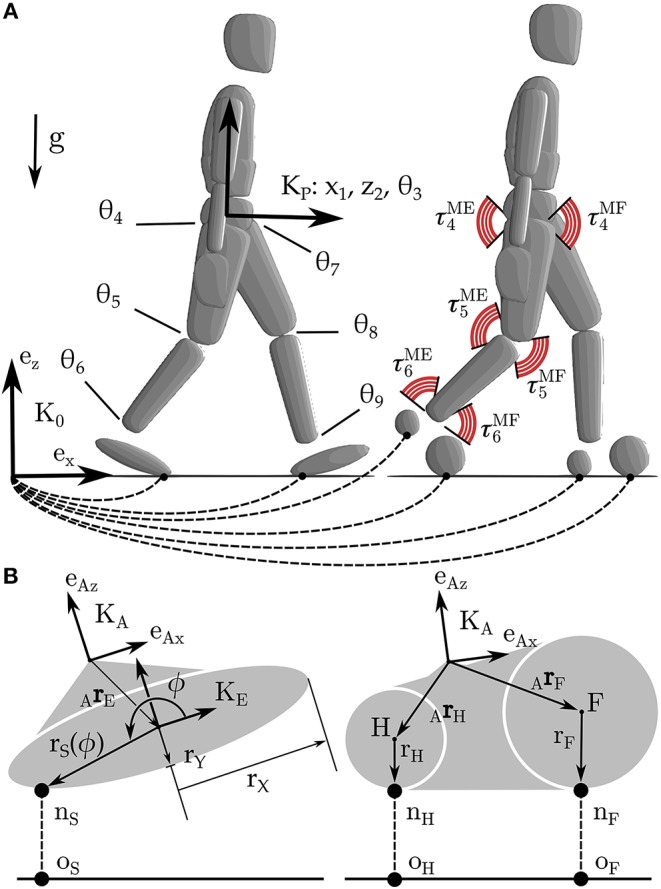
The human body is modeled using a 9 DoF rigid body model in which the hip, knee, and ankle are actuated by pairs of extensor and flexor MTGs **(A)**. The two sagittal plane models are identical except for the foot-ground contact model. In one case foot-ground contact is modeled as a kinematic constraint between an ellipse and a plane, in the other between a pair of circles and a plane **(B)**.

We model the human body as a planar floating base rigid-body system (Figure 1A) which interacts with the ground through one of two different rigid foot ground contact models (Figure 1B). Foot ground interaction is modeled using contact and rolling constraints between an ellipse and a plane, and also between a pair of circles and a plane. The human body models used to test each foot are identical and have seven segments, nine degrees-of-freedom (DoF), and are driven by six pairs of agonist-antagonist muscle-torque-generators (MTGs).

The differential-algebraic equations (DAEs) governing this system are described as

(1) $$\begin{array}{rcl} M\left(q\right)\ddot{q}+c\left(q,\dot{q}\right) & = & \tau +G{\left(q,\dot{q}\right)}^{T}\lambda \end{array}$$

(2) $$\begin{array}{rcl} g_{V}\left(q,\dot{q}\right) & = & 0 \end{array}$$

where *q*, $\dot{q}$, and $\ddot{q}$ are the generalized positions, velocities, and accelerations of the model; *M*(*q*) is the mass matrix, and $c\left(q,\dot{q}\right)$ is the vector of Coriolis, centripetal, and gravitational forces. The kinematic constraints between the foot and the ground are in the vector $g_{V}\left(q,\dot{q}\right)$, while the generalized forces these constraints apply to the system are contained in the term $G{\left(q,\dot{q}\right)}^{T}\lambda$ where $G\left(q,\dot{q}\right)$ is the Jacobian of the constraint equations $g_{V}\left(q,\dot{q}\right)$ with respect to $\dot{q}$ and λ is a vector of Lagrange multipliers. The foot ground constraints, $g_{V}\left(q,\dot{q}\right)$, are described at the velocity level, index-reduced, and applied at the acceleration level.

Throughout this work we indicate position vectors using *r*, direction vectors with *e*, frames with K (which are composed of a position vector to the origin and a rotation matrix), points using letters, linear velocity with *v*, and angular velocities with ω. Forces are denoted using *f* while functions are indicated with f and appear with an argument. Subscripts are used with direction vectors and frames to provide additional information, while a more elaborate system is used with kinematic vectors: the origin of the vector appears in the left subscript, the termination in the right subscript, and the frame the vector is resolved into (if necessary) is indicated in the left superscript. Thus _A_*r*_H_ means the position vector that begins at point A and terminates at point H but is not yet resolved into any particular frame since the left superscript is blank.

The constraints between the ellipse and the plane are applied at the point-of-closest approach (*n*_S_) and are described at the velocity-level using a contact constraint

(3) $${\left(v_{\text{A}}+\omega_{\text{A}}\times \left({}_{\text{A}}r_{\text{E}}+{\text{r}}_{\text{S}}\left(\phi \right)e_{\phi }\right)\right)}^{\text{T}}e_{\text{Z}}=0$$

and a rolling constraint

(4) $$\begin{array}{l} {\left(v_{\text{A}}+\omega_{\text{A}}\times \left({}_{\text{A}}r_{\text{E}}+{\text{r}}_{\text{S}}\left(\phi \right)e_{\phi }\right)\right)}^{\text{T}}e_{\text{X}}=0 \end{array}$$

where *v*_A_ is the linear velocity of the origin of the ankle frame $K_{\text{A}}$, ω_A_ is angular velocity of $K_{\text{A}}$, _A_*r*_E_ is the vector from $K_{\text{A}}$ to the center of the ellipse, *e*_ϕ_ the direction vector that points to *n*_S_, and r_S_(ϕ) is the radius of the ellipse at the polar angle ϕ. Since there is no closed form equation for the point of closest approach between an ellipse and a plane, we numerically solve for ϕ using first the bisection method, and finally Newton's method to polish the root to high accuracy. The parameters of the ellipse-foot (*p*_E_) are defined by the offset of the ellipse from the ankle (_A_*r*_E_), its relative orientation to the angle frame (_A_θ_E_), and the lengths of the major and minor axes of the ellipse (*r*_X_ and *r*_Y_).

The double circle foot contact model uses one of three different sets of constraint equations depending on which circle is in contact with the ground. During heel contact the constraint equations are

(5) $${\left(v_{\text{A}}+\omega_{\text{A}}\times \left({}_{\text{A}}r_{\text{H}}-r_{\text{H}}e_{\text{Z}}\right)\right)}^{\text{T}}e_{\text{Z}}=0$$

(6) $${\left(v_{\text{A}}+\omega_{\text{A}}\times \left({}_{\text{A}}r_{\text{H}}-r_{\text{H}}e_{\text{X}}\right)\right)}^{\text{T}}e_{\text{Z}}=0$$

where *r*_H_ is the radius of the heel circle. During forefoot contact the constraint equations are given by

(7) $${\left(v_{\text{A}}+\omega_{\text{A}}\times \left({}_{\text{A}}r_{\text{F}}-r_{\text{F}}e_{\text{Z}}\right)\right)}^{\text{T}}e_{\text{Z}}=0$$

(8) $${\left(v_{\text{A}}+\omega_{\text{A}}\times \left({}_{\text{A}}r_{\text{F}}-r_{\text{F}}e_{\text{Z}}\right)\right)}^{\text{T}}e_{\text{X}}=0$$

where *r*_F_ is the radius of the forefoot contact. These equations are nearly identical to the constraints used for the ellipse, but without the extra computational expense incurred in computing the point of closest approach. To ensure that the foot is not over-constrained when both circles touch the ground, we apply the contact and rolling constraints of one circle, while the other is constrained with just a contact constraint. The parameters of the double-circle foot (*p*_C_) are defined by the offset of each circle from the ankle (_A_*r*_H_ and _A_*r*_F_), and the radius of each circle (*r*_H_ and *r*_F_).

The model is actuated by six pairs of agonist-antagonist MTGs each of which model groups of extensors and flexors that cross the hip, knee, and ankle. The torque τ^M^ developed by a single MTG resembles that of a rigid-tendon Hill-type muscle model (Zajac, 1988; Millard et al., 2013) and is given by

(9) $$\tau^{\text{M}}=\pm \tau_{\text{o}}^{\text{M}}\left(\text{A}{\text{f}}^{\text{A}}\left(\theta \right){\text{f}}^{\text{V}}\left(\omega \right)\right)$$

where $\tau_{\text{o}}^{\text{M}}$ is the maximum active isometric torque of the MTG, *a* represents the chemical activation of the MTG, f^A^(θ) is the active-torque-angle characteristic, f^ V^(ω) is the torque-angular-velocity curve, and the sign is set to be consistent with the anatomy of the muscle group and the generalized coordinates used to describe the model. The parameters ($\tau_{\text{o}}^{\text{M}}$) and curves [f^A^(θ) and f^ V^(ω)] that define the flexors and extensors of the hip, knee, and ankle are fitted to the data of Anderson et al. (2007) and Jackson (2010). Please see Millard et al. (2017) for a more detailed description of the formulation and parameters of the MTGs. Since walking does not typically stretch the leg muscles appreciably (Arnold and Delp, 2011), we ignore the passive forces developed by the parallel element.

Although it is conventional to describe activation dynamics using an ordinary differential equation with a discontinuity, this formulation is not compatible with gradient-based optimal control methods which require $C_{2}$ continuity. Here we describe activation dynamics using a $C_{2}$ approximation

(10) $$\begin{array}{r} ȧ=\frac{e-\text{A}}{\frac{1}{2}\left(\tau_{\text{A}}+\tau_{\text{D}}\right)} \end{array}$$

where *e* is the excitation signal, *a* is the activation of the muscle. The activation τ_A_ and deactivation τ_D_ time constants are 15 and 50ms, respectively (Thelen, 2003).

At each leg joint the net torque is given by

(11) $$\begin{array}{r} \tau_{i}=\tau_{i}^{\text{MF}}+\tau_{i}^{\text{ME}}-\beta \omega_{i}. \end{array}$$

where F and E designate the joint's flexors and extensors, and β is light passive damping introduced by the musculature and tissue surrounding the joint. The damping coefficient is defined as

(12) $$\begin{array}{r} \beta =\eta \frac{\tau_{\text{o}}^{\text{MF}}+\tau_{\text{o}}^{\text{ME}}}{\omega_{\text{max}}^{\text{MF}}+\omega_{\text{max}}^{\text{ME}}} \end{array}$$

so that the amount of damping is proportional to the strength the musculature, the scaling factor η, and inversely proportional to the maximum angular velocity of the musculature. We use a value of 2.0 for η which results in damping coefficients which range between 2.7 and 7.3 Nms/rad.

We use the open-source dynamics library *Rigid Body Dynamics Library*^1^ (RBDL), an implementation of Featherstone's order-n dynamics methods (Featherstone, 2014), developed by Felis (2016), to solve the forward dynamics of our model. To simulate the MTGs, we use RBDL's muscle model library developed by Millard et al. (2017).

## 3. Evaluation Procedure

As is typical, we first evaluate each foot model in isolation by considering how accurately each tracks the kinematics of the ankle and CoP of a subject's foot. Next, we pair each candidate foot model with a musculoskeletal model and solve a tracking OCP to determine if the experimental subject's gait is in the solution space of each foot model. Finally, we solve a prediction OCP to examine how well each foot performs when it is not guided by experimental data and is free to move.

### 3.1. Experimental Data

The experimental data used in this study comes from a walking trial recorded in an experiment described in Millard et al. (2017). Briefly, the motions and ground forces of a 35-year old male subject (mass 81.7 kg and height of 1.72 m) wearing light hiking shoes were recorded during level walking. OptoTrack IRED markers clusters were used to track the 3-dimensional (3D) movements of 14 body segments (head, upper-torso, mid-back, pelvis, thighs, shanks, feet, upper-arms, and lower arms) while Kistler force plates (Kistler GmbH, Germany) were used to measure ground forces. The recordings were conducted at Vrije Universiteit Amsterdam according to the guidelines of the Declaration of Helsinki 2013, approved by the ethics committee in Faculteit der Gedrags- en Bewegingswetenschappen (Faculty of Behavioral and Movement Sciences), and with written and informed consent from the subject. Mass and inertia properties were computed using Zatsiorsky's regression equations (Zatsiorsky, 2002) while the geometry of the human model is extracted using digitized bony landmarks from the experimental subject.

### 3.2. Foot Model Fitting

**Figure 2:**
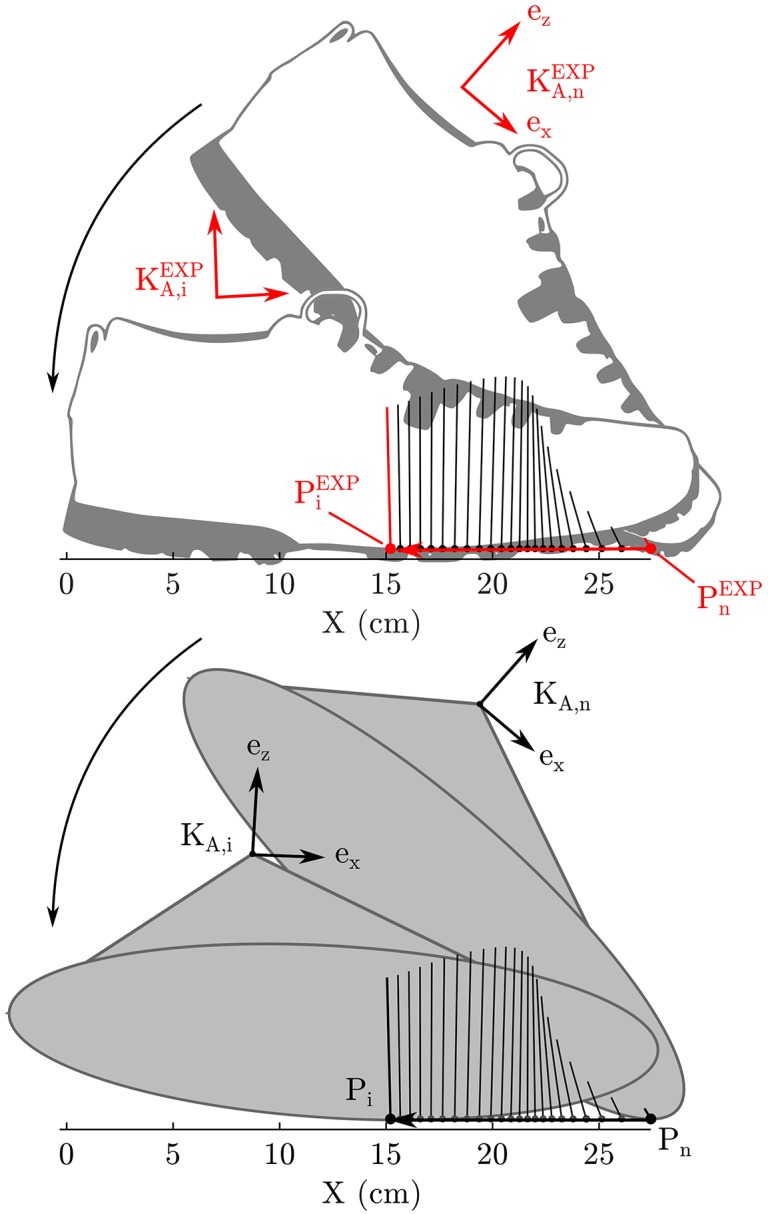
The error in the geometry of each foot model is fitted by posing the foot model so that its contact point and foot orientation matches the subject's at toe-off. Next the foot model is rolled backwards (without slipping) so that it's contact point matches the subject's recorded CoP. This process is continued until heel strike. The geometric error of the foot model is the weighted sum of position and orientation errors between the model's ankle frame and the subject's ankle frame.

As is typical in the literature, first we will fit the foot model in isolation before proceeding to use it with the whole body model. Since the force plates very accurately record the CoP trajectory, we have elected to fit the foot model by precisely matching the recorded CoP kinematics (experimental quantities are denoted with ^EXP^) and then measuring the error between the position and orientation of the $K_{\text{A}}$ and the $K_{\text{A}}^{\text{EXP}}$ frame (Figure 2). Prior to fitting the foot model, the data used for fitting was segmented to only include samples in which the normal contact force was >5% of the peak recorded ground force (928N). To fit the model foot, it was initially posed at the same orientation as the subject's foot had at toe-off (at time sample n) and offset so that the contact point of the model *r*_P, n_ coincided with the recorded CoP $r_{\text{P},\text{n}}^{\text{EXP}}$. Next, the model foot was rolled without slipping backwards until the foot contact point of the model matched the recorded CoP, a process which continued until the time of heel strike was reached (at time sample 1). We elected to pose the model in the toe-off position and roll it backwards because this made it easier for us to manually find a good set of initial parameters prior to beginning the optimization.

The geometric parameters of each foot model (*p*_E_ and *p*_C_) are fitted by minimizing the cost function

(13) $$\begin{array}{r} \min\overset{1}{\underset{i=n}{\sum }}\frac{{\left(f_{\text{P},\text{i}}^{\text{EXP}}\right)}^{\text{T}}e_{\text{Z}}}{\max\left({\left(f_{\text{P}}^{\text{EXP}}\right)}^{\text{T}}e_{\text{Z}}\right)}\left(w_{\text{r}}{\left(r_{\text{A},i}-r_{\text{A},\text{i}}^{\text{EXP}}\right)}^{2}+w_{\alpha }{\left(\alpha_{\text{A},\text{i}}-\alpha_{\text{A},\text{i}}^{\text{EXP}}\right)}^{2}\right), \end{array}$$

where the vectors *r*_A_ and α_A_ are the origin and orientation of the ankle frame, and $f_{\text{P},\text{i}}^{\text{EXP}}$ is *i*th experimentally recorded ground reaction force vector. The cost of each sample *i*, is weighted by the normalized magnitude of the contact force so that the final fit is closest when the ground forces are highest. The weights *w*_r_ and *w*_α_ are set to (1/0.01)^2^ and ${\left(1/\left(\frac{1}{4}\pi \right)\right)}^{2}$ so that distances on the order of 1 cm and $\frac{1}{4}\pi$, which we consider to be large errors in this context, result in an error value of 1. We did not allow all parameters to vary but fixed the total length of the foot model to be 30.5 cm, which is 0.5 cm longer than the light hiking shoes worn by the subject. This extra constraint is added to prevent the optimization routine from converging on an unrealistically short foot. Though a shorter foot may fit the kinematics of this test best, it causes problems when the subject's CoP is followed in the tracking OCP problem (described in section 3.4).

The resulting prediction problem for the ellipse-foot has only four optimization variables (the center and orientation of the ellipse and the radius of the minor axis) while the double-circle foot has five parameters (the radius of both circles, the center location of the forefoot circle, and the height of the center of the hind-foot circle). In each case the least squares problem is initialized using manually and then solved using a Nelder-Mead simplex method (Nelder and Mead, 1965; Lagarias et al., 1998) to a tolerance of 10^−6^ (MATLABR2018a). Each model is evaluated based on the how closely the ankle frame of the model tracks the subject's ankle movements.

### 3.3. Walking as an Optimal Control Problem

In this work we use two different types of OCPs: a tracking problem which tries to follow experimental data, and a prediction problem that tries to minimize a cost function. Both of these OCPs define walking using the same mathematical framework, and differ only in the cost function used and a few constraints. Here we describe how walking is defined as optimal control problem in general before proceeding to describe the specific differences between the tracking and prediction OCPs.

An OCP has the objective of identifying the vector of state *x*(·) trajectories, control *u*(·) trajectories, and constant parameters *p*, that minimize the sum of the Lagrange ϕ^L^ and Mayer Φ^M^ terms in the cost function across *n*_*p*_ distinct phases

(14) $$\begin{array}{r} \underset{x\left(\cdot \right),u\left(\cdot \right),\nu }{\min}\sum_{j=0}^{n_{p}-1}\left(\overset{\nu_{j+1}}{\underset{\nu_{j}}{\int }}\phi_{j}^{\text{L}}\left(x\left(t\right),u\left(t\right),p\right)dt\right)+\overset{n_{p}-1}{\underset{j=0}{\sum }}\Phi_{j}^{\text{M}}\left(x\left(t\right),u\left(t\right),p\right) \end{array}$$

where *j* iterates sequentially across the phases that begin at time ν_*j*_ and terminate at time ν_*j*+1_. In addition to minimizing the cost function, the state trajectories must satisfy the state derivatives and impact state transitions

(15) $$\begin{array}{rcl} ẋ\left(t\right) & = & f_{j}\left(t,x\left(t\right),u\left(t\right),p\right), \end{array}$$

(16) $$\begin{array}{r} x\left(t_{j}^{+}\right)=c_{j}\left(x\left(t_{j}^{-}\right)\right),\;\text{for}t\in \left[\nu_{j-1},\nu_{j}\right],\\ \;j=1,\ldots ,n_{p},\nu_{0}=0,\nu_{n_{p}}=T. \end{array}$$

which take the form of the DAEs in Equations (1, 2), and activation dynamics of the MTGs in Equation (10). In this work, state transitions from foot impacts cause discrete changes in the generalized velocities of the model but affect no other states.

The state vector, $x=\left(q,\dot{q},\text{A}\right)$, of the musculoskeletal model has 30 states, 18 of which correspond to generalized positions and velocities while the additional 12 come from the vector of muscle activations. The vector of control signals, *u*(·), is composed of the twelve excitation signals that affect the activation dynamics of the MTGs as described in Equation (10). The vector of generalized forces has 6 non-zero elements, τ = (0, 0, 0, τ_4_, τ_5_, τ_6_, τ_7_, τ_8_, τ_9_), corresponding to torques that the MTGs and passive damping apply to each joint. The leading three entries in τ are zero because there are no generalized forces acting between the inertial frame and the pelvis. The number of kinematic constraints applied to the model ranges between 2 and 6 depending on the foot-model being simulated and the constraint set that is active.

**Figure 3:**
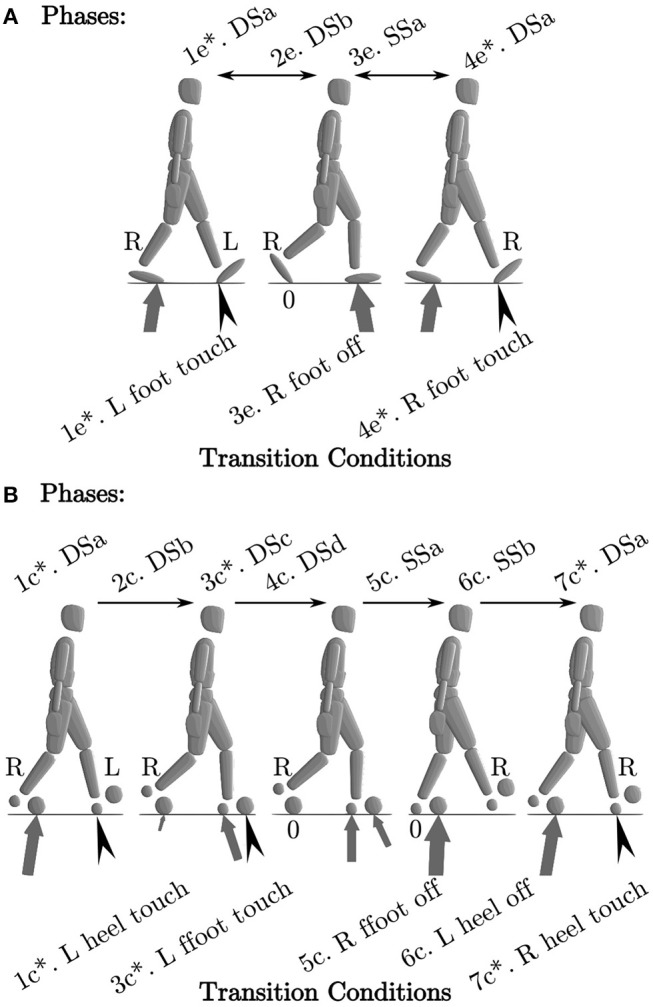
We model walking as a multi-phase process. The ellipse-foot results in a four-phase definition of walking **(A)**, while the double-circle foot (with its separate hind and forefoot contacts) results in a 7 phase definition of walking **(B)**. For brevity we refer to the ellipse model with an “e”, the double circle model with a “c”, double stance with (DS), and single-stance with (SS). Black chevrons indicate an impact occurs. An “*” indicates that the phase is instantaneous.

We formulate walking as a multi-phase OCP that has four phases for the ellipse-foot (Figure 3A), and seven phases with the double-circle foot (Figure 3B). To distinguish between the various phases and foot model we introduce a number of short forms: ellipse-foot (e), double-circle foot (c), double-stance (DS), single-stance (SS), and instantaneous phases are marked with an “^*^.” Walking using the ellipse-foot consists of two continuous phases and two instantaneous phases:

- 1e^*^. DSa occurs when the left foot touches the ground;
- 2e. DSb: is a double stance phase;
- 3e. SSa: is a continuous single-stance phase that begins when the right foot's ground force goes to zero;
- 4e. DSa: is identical to 1e^*^ but with the left and right legs mirrored.

The double-circle foot requires four continuous phases and three instantaneous phases to describe walking:

- 1c^*^. DSa: occurs when the left heel circle touches the ground;
- 2c. DSb: a double-stance phase between the right forefoot and left heel;
- 3c^*^. DSc: occurs when the left forefoot touches the ground;
- 4c. DSd: a double-stance phase between the right forefoot, left heel, and left forefoot;
- 5c. SSa: a single-stance phase with left heel and forefoot on the ground;
- 6c. SSb: a single-stance phase with the left forefoot on the ground;
- 7c^*^. DSa: is identical to 1c^*^ but with the left and right legs mirrored.

In both cases the foot-ground impulse are stored in the vector Δ. Note that these specific phase descriptions match the experimental subject's gait, but there are many other possible phase descriptions for the double-circle foot.

We use continuous constraints

(17) $$\begin{array}{r} 0\leq g_{j}\left(t,x\left(t\right),u\left(t\right),p\right)\;\text{for}t\in \left[\nu_{j-1},\nu_{j}\right] \end{array}$$

on state and control bounds, as well as using phase specific constraints. The bounds on *q* are set to be at least ±1m and (for the linear coordinates) and ±1 radian (for the angular coordinates) larger than the experimental measurements (except in knee extension where 0.1 radian of hyper-extension is permitted). Similarly the bounds on $\dot{q}$ are set to be at least ±1m/s and ±1rad./s larger than the experimental measurements. The vectors *a*(·) and *u*(·) are constrained to be between zero and one. Phases which begin with an impact include equality constraints at the position level so that the respective foot-ground constraints begin on the constraint manifold. During swing phases an inequality constraint is used to ensure that the swing foot does not touch the ground. All ground forces and impulses are constrained act unilaterally and have tangential components that are limited by the coefficient of friction which we assume to be 0.8. To ensure that the final solution represents periodic and symmetric walking we apply periodicity constraints so that the joint angles, activations, and ground forces of the right leg (left leg) in the initial phase match the corresponding values of the left leg (right leg) in the final phase. At the velocity level we apply periodicity constraints to the linear and angular velocity of the pelvis.

### 3.4. Least-Squares Tracking Problem

To determine if the subject's gait is within the solution space of the model we form a least-squares OCP to track the subject's data. As previously noted, not all of the experimental measurements are of equal accuracy: while the CoP and ground forces are very accuracy measured by the force plates, the kinematic data is subject to error on the order of a centimeter or two due to skin artifact (Fuller et al., 1997). To make the best use of the data, we have formulated a tracking (indicated by _T_ where appropriate) problem which has a Lagrange $\phi_{\text{T}}^{\text{L}}$ and Mayer $\Phi_{\text{T}}^{\text{M}}$ terms of

(18) $$\begin{array}{l} \phi_{\text{T}}^{\text{L}}=\sum_{i=3}^{9}{\left(w_{\theta ,i}(\theta_{i}-\theta_{i}^{\text{EXP}}\right)}^{2}+w_{\omega ,i}{\left(\omega_{i}-\omega_{i}^{\text{EXP}}\right)}^{2})\\ \;+w_{\text{P}}{\left(r_{\text{P}}-r_{\text{P}}^{\text{EXP}}\right)}^{2}+w_{\text{F}}{\left(f_{\text{P}}-f_{\text{P}}^{\text{EXP}}\right)}^{2}+10^{-3}\sum_{i=0}^{n_{u}}\left(u_{i}^{2}+{\text{A}}_{i}^{2}\right) \end{array}$$

(19) $$\begin{array}{rc} \Phi_{\text{T}}^{\text{M}} & =10^{-5}\overset{n_{\Delta }}{\underset{i=0}{\sum }}\Delta_{i}^{2} \end{array}$$

where *n*_*u*_ and *n*_Δ_ are the number of control signals and the number of impulses respectively. This cost function is applied across all phases of the problem. Note that the Lagrange term is an integrated quantity, as such all of the experimental data is interpolated as a function of time prior to evaluating and numerically integrating (Equation 18).

The Lagrange term is formulated so that the angles and angular velocities of the pelvis and leg joints (indices for θ_3_−θ_9_ illustrated in Figure 1) are tracked along with the CoP , and ground forces. The weighting terms on the angles *w*_θ, *i*_, and angular velocities *w*_ω, *i*_ are set to $\frac{1}{\pi /4}$ and $\frac{0.1}{\pi /4}$, respectively with the exception of the ankle joint which is set to $\frac{1}{100}$ of these nominal values: kinematic error that the foot introduces will be most readily observed at the ankle. The weighting terms *w*_P_ and *w*_F_ associated with the normal components are normalized with respect to maximum recorded contact forces. In addition, we have introduced three regularization terms: the sum of squared control signals *u*^2^ and activations *a*^2^ in the Lagrange term, and the sum of squared ground impulses Δ^2^ in the Mayer term. The coefficient on the regularization terms has been chosen so that the terms have a similar magnitude. Here we evaluate the Lagrange term only at the shooting nodes (making this a discrete least-squares problem).

### 3.5. Minimization Prediction Problem

Inspired by the experimental work of Hoyt and Taylor (1981) and later Farley and Taylor (1991), we formulate a prediction (indicated with a _P_) cost function in with a Lagrange term on muscle activation

(20) $$\begin{array}{r} \phi_{\text{P}}^{\text{L}}=\overset{n_{u}}{\underset{i=0}{\sum }}{\text{A}}_{i}^{2} \end{array}$$

and a Mayer term that includes foot-ground impacts

(21) $$\begin{array}{r} \Phi_{\text{P}}^{\text{M}}=w_{\Delta }\overset{n_{\Delta }}{\underset{i=0}{\sum }}\Delta_{i}^{2}. \end{array}$$

Here *w*_Δ_ is set to 10^−2^, a value which found in our preliminary simulations to be sufficient to reduce the ground force discontinuities introduced by ground impacts to levels consistent with the experimental data. So that the physical demands placed on the foot models are comparable to the subject data, in addition, we introduce two constraints: that the average forward velocity of the solution is identical to the subject's (1.01 m/s), and that the step length of the model matches that of the subject (0.61 m). Note that this problem formulation, while useful for our purposes, cannot be used to predict human walking in general because we have explicitly included a desired forward velocity and step length.

### 3.6. Numerical Solution Method

To solve the tracking and prediction OCPs specified we use a direct multiple shooting method described by Bock and Pitt (1984) and implemented in the software package MUSCOD-II developed by Leineweber et al. (2003). In a direct approach, the infinite-dimensional space of control functions *u*(·) is discretized in time using functions which provide only local support. Here we use piece-wise linear functions to describe the excitation signals to the MTGs. State parameterization is performed by the multiple shooting technique which transforms the OCP, together with the control discretization, from an infinite dimensional problem into a finite dimensional problem which is then solved iteratively using a sequential-quadratic-programming (SQP) solver that has been tailored to exploit the structure of the problem.

We initialize the problem with a rough initial solution: positions and velocities are initialized using a linear interpolation of the experimental positions which are then polished to satisfy the foot ground constraints, activations are set to 0.01, control signals are set to 0.025. The initial solution does not satisfy the OCP constraints and is not a feasible motion. The OCPs using the ellipse and double circle foot models are discretized using 25 and 31 shooting nodes and control intervals, respectively. Each shooting interval is integrated using the Runge-Kutta-Fehlberg method with an absolute and relative tolerance of 10^−8^. Note that, in contrast to direct-collocation (Von Stryk, 1993), the dynamics of the system are simulated using a variable-step integrator over the entire duration of the simulation. To reduce the drift of the foot-ground constraints, we use Baumgarte stabilization (Baumgarte, 1972) applied to the contact constraint position and velocity errors, and to the rolling constraint velocity errors. The OCPs are run until the Karush-Kuhn-Tucker condition is satisfied to a tolerance of 10^−5^. Each problem required between 20 and 50 min of processing time on an Intel i7-3630QM CPU with a clock speed of 2.40 GHz.

## 4. Results

**Figure 4:**
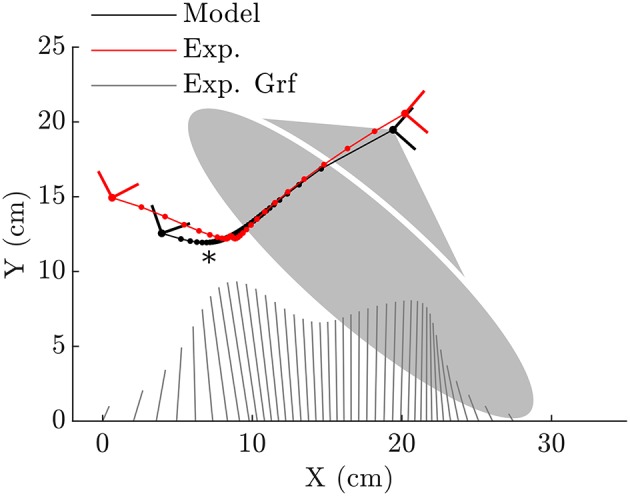
When the ellipse-foot is constrained to track the CoP (illustrated with the butterfly plots for reference) from the subject's data it is able to closely reproduce the subject's foot movements during mid-stance and for most of toe-off. The initial kinematics of the foot during heel contact are not well-captured. The continuous rolling motion of the foot forces the ankle frame through a smoothened cusp which is annotated with an “^*^”. Note that the dots which appear on each line would coincide if the foot model perfectly fit the subject's foot movements.

**Figure 5:**
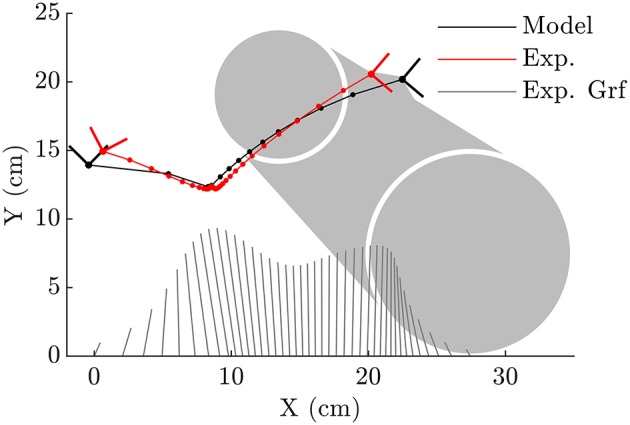
The double-circle foot is able to capture the subjects ankle kinematics during initial heel contact better than the ellipse-foot, though it has difficulty tracking the points between heel contact and mid-stance. During mid-stance both the heel and forefoot contacts touch the ground which fixes the ankle at the same location. Note that the dots which appear on each line would coincide if the foot model perfectly fit the subject's foot movements.

When forced to track the recorded CoP, both the fitted ellipse-foot and the double-circle foot produce ankle trajectories that differ from the subject's on the order of one centimeter, but have maximum errors that exceed this desired limit as shown in Table 1. Though the fitting process restricted the length of the foot models to have a realistic length, the height of the foot model shapes does exceed the size of a shoe particularly at the forefoot of the double-circle foot as shown by the parameter value for *r*_F_ shown in Table 2. The double-circle foot offers a slightly better tracking of the subject's ankle position while the ellipse-foot is slightly superior in its reproduction of the orientation of the ankle frame. The ankle trajectory (Figures 4, 5) traced by the two different foot models show that the highest errors occur during heel contact: it is during this period that the rigid approximation to the foot is worst because the heel pad and shoe are compressing. Further, the ankle trajectory of the two models displays a characteristic difference: the ellipse foot produces a u-shaped ankle trajectory (marked with a “^*^” in Figure 4) while the double circle produces a v-shaped trajectory due to the forefoot circle plastically contacting the ground.

**Table 1:** The position ($r_{\text{A}}-r_{\text{A}}^{\text{EXP}}$) and orientation ($\alpha_{\text{A}}-\alpha_{\text{A}}^{\text{EXP}}$) errors between the ankle frame of each respective foot model and experimental data of a subject's foot during the stance phase.

	**Ellipse**	**Circle-circle**
**ε(*t*)**	**μ(|ε(***t***)|_**2**_)±σ(|ε(***t***)|_**2**_)**	
$r_{\text{A}}-r_{\text{A}}^{\text{EXP}}$	1.1 ± 1.1 cm	0.7 ± 0.9 cm
$\alpha_{\text{A}}-\alpha_{\text{A}}^{\text{EXP}}$	4.7°± 3.1°	7.1°± 2.8°
ε(*t*)	max(|ε(*t*)|_2_)	
$r_{\text{A}}-r_{\text{A}}^{\text{EXP}}$	4.4 cm	4.3 cm
$\alpha_{\text{A}}-\alpha_{\text{A}}^{\text{EXP}}$	10.6°	18.1°

*These differences are present when each foot model is constrained to have a CoP which is identical to that of the subject*.

**Table 2:** The parameters of each foot model which best fit the subject's data are listed below.

**Ellipse foot**	
${}_{\text{A}}^{\text{A}}r_{\text{E}}$	(4.30, −8.11)
_A_α_E_	0
*r*_X_	15.25
*r*_Y_	4.03
**Double circle foot**	
${}_{\text{A}}^{\text{A}}r_{\text{H}}$	(−6.19, −6.64)
*r*_H_	4.87
${}_{\text{A}}^{\text{A}}r_{\text{F}}$	(11.97, −6.56)
*r*_F_	7.46

*As noted in section 3.2, the heel segment is placed so that the length of the foot is 30.5 cm, while all other parameters are free to vary. Note that _A_α_E_ is the rotation from $K_{\text{A}}$ to $K_{\text{E}}$. Please see Figure 1 for a graphical depiction of the remaining parameters*.

**Figure 6:**
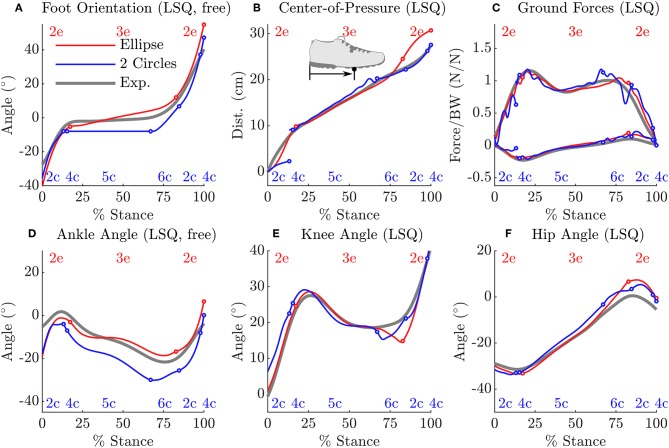
A comparison between the tracking OCP solutions from the ellipse-foot and double-circle foot models and the subject's data. Transitions between phases are indicated using a circle mark, while the labels for the continuous phases (described in section 3.3) appear at the top (ellipse-foot in red) and bottom (double-circle foot in blue) of each plot. Note that the ankle angle **(D)** [and thus orientation of the foot **(A)**] is weakly tracked because kinematic errors at this joint are scaled to be $\frac{1}{100}$ the value of other tracked quantities. The ellipse-foot uniformly tracks the subject's data with a higher degree of accuracy than the double-circle foot. Both models have difficulty tracking the subject's knee angle at 75% stance **(E)**, and the subject's ground forces near 25% and 75% of stance. During heel-only contact the double-circle foot is not able to track the subject's CoP movements **(B)**, but closely follows the subject's CoP trajectory thereafter. Due to ground impacts both models have ground forces that begin the stance phase with finite values. The double-circle foot has an additional discontinuity in both the CoP profile **(B)** and ground force profile **(C)** when the forefoot strikes the ground. The forefoot strike completely arrests the angular velocity of the double-circle foot (see the flat line in phases 4c and 5c in **A**) while the ellipse-foot continuously rotates during stance. Both solutions are able to closely follow the subject's hip angle trajectory **(F)**.

The solution of the tracking OCP shows that the ellipse-foot is able to reproduce the orientation of the subject's foot (Figure 6A), and ankle angle (Figure 6D) with better accuracy than the double-circle foot as the summary statistics show in Table 3. Both tracking OCPs had difficulty reproducing the subject's knee angle (Figure 6E) between near 75% of the stance phase, because the foot models fail to capture the shape of the foot at the transition between mid-stance and toe-off. The hip angle is tracked with comparable accuracy by both foot models (Figure 6F and Table 3). Though the double-circle foot tracks the CoP more accurately than the ellipse-foot (Figure 6B and Table 3), the ground forces produced by the double-circle foot exhibit oscillations that are present to a lesser degree in the ellipse-foot (Figure 6C).

**Table 3:** The average and maximum errors between the subject's stance foot and that of the tracking solution show that the ellipse-foot results in a better replication of the subject's gait than the double-circle foot, particularly at the ankle.

	**Ellipse**	**Circle-circle**
**ε(*t*)**	**μ(|ε(***t***)|_**2**_)±σ(|ε(***t***)|_**2**_)**	
$\alpha_{\text{A}}-\alpha_{\text{A}}^{\text{EXP}}$	3.6°± 2.6°	5.4°± 2.1°
$r_{\text{P}}-r_{\text{P}}^{\text{EXP}}$	1.5 ± 1.6 cm	0.8 ± 1.0 cm
${\left(f_{\text{P}}-f_{\text{P}}^{\text{EXP}}\right)}^{\text{T}}e_{\text{X}}$	30.0 ± 17.5 N	29.2 ± 23.7 N
${\left(f_{\text{P}}-f_{\text{P}}^{\text{EXP}}\right)}^{\text{T}}e_{\text{Z}}$	46.8 ± 38.1 N	69.6 ± 58.9 N
$\theta_{\text{A}}-\theta_{\text{A}}^{\text{EXP}}$	3.1°± 2.1°	7.3°± 2.1°
$\theta_{\text{K}}-\theta_{\text{K}}^{\text{EXP}}$	1.6°± 1.7°	2.2°± 1.8°
$\theta_{\text{H}}-\theta_{\text{H}}^{\text{EXP}}$	2.9°± 2.5°	3.0°± 1.5°
**ε(*t*)**	**max(|ε(*t*)|_2_)**	
$\alpha_{\text{A}}-\alpha_{\text{A}}^{\text{EXP}}$	14.6°	8.5°
$r_{\text{P}}-r_{\text{P}}^{\text{EXP}}$	4.7 cm	5.4 cm
${\left(f_{\text{P}}-f_{\text{P}}^{\text{EXP}}\right)}^{\text{T}}e_{\text{X}}$	74.7 N	130.2 N
${\left(f_{\text{P}}-f_{\text{P}}^{\text{EXP}}\right)}^{\text{T}}e_{\text{Z}}$	128.4 N	266.1 N
$\theta_{\text{A}}-\theta_{\text{A}}^{\text{EXP}}$	13.9°	12.7°
$\theta_{\text{K}}-\theta_{\text{K}}^{\text{EXP}}$	6.4°	6.9°
$\theta_{\text{H}}-\theta_{\text{H}}^{\text{EXP}}$	7.5°	6.0°

*Note that the ankle angle and foot orientation are free to vary, while all other quantities listed below are tracked*.

**Figure 7:**
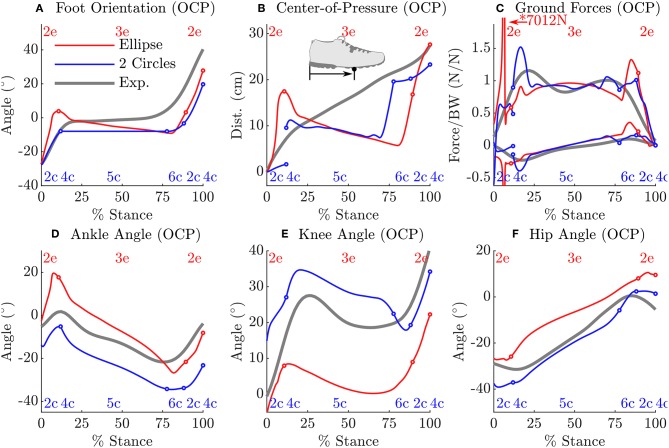
A comparison between the prediction OCP solutions from the ellipse-foot and double-circle foot models and the subject's data. As in Figure 6, phase transitions are marked with a circle, continuous phase labels appear at the top (ellipse-foot in red) and bottom (double-circle foot in blue) of each plot. Though both solutions differ from the subject's data, both solutions also differ from each other: the shape of the foot has a large influence on the kinematics of the ankle **(D)**, knee **(E)**, and hip **(F)** of the stance leg. In one regard the solutions of the ellipse-foot and double-circle foot are similar: in both cases the model keeps its weight on its heel until the last moments of the stance phase **(B)**. Note that the double-circle foot produces ground forces that are similar in magnitude to the tracking problem while the ellipse-foot produces a large transient ground force **(C)** shortly after contact within the continuous phase 2e. This transient is not due to an impact, nor constraint stabilization, but instead due to an interaction between the walking motion and the ellipse-foot model. As with the tracking solution, the angular velocity of the double-circle foot is arrested between phases 4c and 5c by the forefoot contact while the ellipse-foot continuously rotates during stance **(A)**. Finally note that phase 4c, while of brief duration in the tracking solution, is of zero duration in the prediction solution.

The solutions of the prediction OCP from each model deviates from the subject's data in general as shown in Table 4, but in different ways as observed at the kinematics of the hip, knee and ankle (Figures 7D–F). These large differences underscore how influential the shape of the foot is on the gait of the model because everything else about the two problems is identical except for the foot model. Another difference of note is observed in the ground forces produced by the ellipse-foot model: the normal and tangential forces exhibit a transient that is about 13 ms in duration that departs from the recorded ground forces by 6582.7 N and 2137.0 N (marked with a “^*^” Figure 7C), respectively. The nature of the transient is not numerical (the largest Baumgarte forces are 10.6 N), nor due to an impact, but due to an interaction between the motion of the model and the single curved foot segment: at precisely this moment the ankle of the ellipse-foot is at the bottom-most part of the u-shaped trajectory it traces (marked with a “^*^” in Figure 4). The ellipse-foot rotates the ankle quickly (hitting the upper bound 14.6 $\frac{r\text{A}d}{s}$) through the u-shaped trajectory accelerating the ankle frame upwards. Since the knee is nearly straight at this time the entire mass of the torso is also accelerated upwards. The brief, but rapid, acceleration of the ankle frame of the ellipse-foot results in a brief, but large, spike in the simulated ground reaction forces. To confirm this suspicion we re-ran the prediction OCP with but limited the angular velocity (from 14.6 $\frac{r\text{A}d}{s}$ to 3.96 $\frac{r\text{A}d}{s}$) of the ankle joint until the peak contact forces were comparable to those of the double-circle foot (1,444 N vs. 1,219 N). Though this extra constraint reduced the unrealistic ground forces, the constraint itself represents a departure from reality because the subject's ankle rotated at a greater velocity (4.41 $\frac{r\text{A}d}{s}$ > 3.96 $\frac{r\text{A}d}{s}$) during the experiment. In contrast, the plastic impact of the forefoot circle arrests the motion of the double-circle foot effectively preventing the force transient produced by the ellipse-foot. Please see the accompanying Supplementary Material section for videos, additional plots, and code for both the models and the OCPs.

**Table 4:** The difference between the results of the prediction OCP of each model's stance leg and that of the subject show that, when free to vary, the final gait is quite different from that of the subject.

	**Ellipse**	**Circle-circle**
**ε(*t*)**	**μ(|ε(***t***)|_**2**_)±σ(|ε(***t***)|_**2**_)**	
$\alpha_{\text{A}}-\alpha_{\text{A}}^{\text{EXP}}$	9.9°± 5.8°	10.7°± 7.7°
$r_{\text{P}}-r_{\text{P}}^{\text{EXP}}$	5.8 ± 5.0 cm	4.1 ± 2.8 cm
${\left(f_{\text{P}}-f_{\text{P}}^{\text{EXP}}\right)}^{\text{T}}e_{\text{X}}$	102.2 ± 212.1 N	40.2 ± 27.5 N
${\left(f_{\text{P}}-f_{\text{P}}^{\text{EXP}}\right)}^{\text{T}}e_{\text{Z}}$	290.1 ± 666.2 N	105.4 ± 102.9 N
$\theta_{\text{A}}-\theta_{\text{A}}^{\text{EXP}}$	8.2°± 4.3°	12.8°± 4.2°
$\theta_{\text{K}}-\theta_{\text{K}}^{\text{EXP}}$	14.6°± 6.3°	7.5°± 3.7°
$\theta_{\text{H}}-\theta_{\text{H}}^{\text{EXP}}$	8.5°± 4.1°	4.2°± 2.4°
**ε(*t*)**	**max(|ε(*t*)|_2_)**	
$\alpha_{\text{A}}-\alpha_{\text{A}}^{\text{EXP}}$	17.0°	22.9°
$r_{\text{P}}-r_{\text{P}}^{\text{EXP}}$	16.3 cm	12.7 cm
${\left(f_{\text{P}}-f_{\text{P}}^{\text{EXP}}\right)}^{\text{T}}e_{\text{X}}$	2137.0 N	144.6 N
${\left(f_{\text{P}}-f_{\text{P}}^{\text{EXP}}\right)}^{\text{T}}e_{\text{Z}}$	6582.7 N	595.6 N
$\theta_{\text{A}}-\theta_{\text{A}}^{\text{EXP}}$	19.4°	20.7°
$\theta_{\text{K}}-\theta_{\text{K}}^{\text{EXP}}$	21.9°	17.7°
$\theta_{\text{H}}-\theta_{\text{H}}^{\text{EXP}}$	15.0°	9.6°

*Although the kinematics of the stance ankle from the ellipse-foot model more closely follow the subject than the double-circle foot, the ellipse-foot has a large force transient (see section 4 for details) due to its mechanics. Though the ground forces created by the double-circle foot model differ from the subject, these errors are relatively small when compared to similar works in the literature*.

## 5. Discussion

While there are many applications for computerized gait prediction, few applications are possible without an accurate model of foot-ground contact. Though much attention has been given to modeling the foot it has proven difficult to simultaneously achieve realistic foot-ground contact kinematics and simulated ground forces. In this work we evaluated two rigid foot-ground contact models in terms of how well each replicated the kinematics of the stance foot and how each affected a tracking OCP and a prediction OCP. The multiple layers of evaluation proved useful. Although the ellipse foot model is better able to fit the kinematics of the stance foot in the least squares OCP, the prediction OCP illustrates that, because this foot model continuously rolls at a single contact point, it is capable of producing enormous contact forces due to the curve it forces the ankle through. This result has a larger implication: the accuracy displayed by a foot-ground contact model during an isolated fitting, or a tracking OCP, does not necessarily generalize to a prediction OCP. In addition, to our knowledge, this is the first work in the literature which solves two prediction OCPs that are identical in every respect except for the model of foot ground contact. The differing predictions of the ellipse-foot and double-circle foot models confirm a long held suspicion that the model of foot-ground contact has a large influence on the optimal motion of the model.

The transient present in the prediction OCP of the ellipse-foot indicates that foot models consisting of a single roll-over shape (Ren et al., 2007; Römer, 2018; Römer et al., 2018) should be treated with some caution. Although the transient we observed with the ellipse-foot does not appear in the work of Ren et al. (2007) there are a few reasons why this might be true. First, Ren et al. (2007) did not allow the feet to move freely during double stance, but constrained the CoP trajectory and ground forces under each foot to follow prescribed linear function. Constraining the movements reduces the magnitude of the simulated ground forces as clearly shown by the tracking solution (Figure 6C) and the prediction OCP with the constrained ankle angular velocity. While the constrained solutions produce more realistic results, this is an undesirable option: it is not clear what the constraint should be ahead of time. The second reason why Ren et al. (2007) may not have observed this transient is because they sampled system dynamics discretely during the solution process: the transient could have been skipped between grid points. Due to the brief nature of the transient, if the model is being simulated using a grid of time points (as is typical of direct-collocation) it is important that a final high-resolution integration be performed to ensure that the results have not been unduly affected.

The optimal control solutions of Römer (2018) also have ground forces which are free of transients (personal communication, ground forces are not reported in the thesis) likely because of differences in the problem formulation and solution method. We have used a forward-dynamics problem formulation which allows the optimization routine to manipulate the generalized forces but then integrates the dynamics of the system forward in time. Römer (2018) made use of a hybrid-zero-dynamic (HZD) approach developed by Westervelt et al. (2007) which uses a mixture of an inverse- and forward-dynamic problem formulation: all of the joints of the model are constrained to follow polynomial functions of the whole-body lean angle; the entire system is reduced to a single DoF which is integrated forward in time. The force transient we observed required a rapid change in the angular velocity of a foot, a rapid change which cannot be described using the polynomials employed by Römer (2018).

The inevitable discrepancies that arise between predicted motions and typical human movement can be illustrative of gaps between our understanding of the mechanics of the body, and how these structures coordinated during movement. Both models resulted in tracking OCP solutions in which the ankle angles which differed from the subject's at heel contact, and the knee angle departed from the subject's near 75% of the stance phase. The most likely explanation for both of these problems is that the shapes we used to represent the foot are a poor match at heel contact and near the transition from mid-stance to toe-off. The large increase in error between the prediction OCP and the experimental data show some obvious directions for improvement. In both cases, the model kept its weight close to its hind foot (Figure 7B) before rapidly pushing-off. This trajectory results in a large error between the simulations and the experimental data of the orientation of the foot (Figure 7A) and the CoP trajectory (Figure 7B). The departure in CoP trajectory is likely due to the fact that the MTGs we used in this work have rigid tendons which do not offer the cost savings that a elastic tendon can when it is loaded slowly and allowed to recoil rapidly. The rapid force oscillations present during the stance phase of the double-circle foot prediction OCP solution, while within the limits of the activation model, are not present in experimental recordings of human walking (Figure 7C). We suspect that these oscillations may be due to the fact that a Hill-model does a poor job of capturing the stiffness and damping properties of actively lengthened muscle (Kirsch et al., 1994). These force oscillations would appear larger with the double-circle foot during heel and forefoot contact because it is constrained from moving and thus perfectly transmits the wrench applied to the ankle to the ground. The ellipse-foot, in contrast, is always free to rotate about its contact point and would move, attenuating the observed ground force oscillation.

## 6. Conclusions

Single segment rigid foot ground contact models are an attractive means to model the foot but should be treated with caution: under the right circumstances these foot models can produce large transient forces if the ankle rapidly moves through a u-shaped trajectory after heel contact. In contrast, we did not observe the same transient using the two-segment rigid foot model because the plastic impact of the forefoot arrests the motion of the ankle through its v-shaped trajectory. Although the two-segmented rigid foot model results in an OCP with substantially more phases than a single segment foot, the two-segmented foot has a benefit: it does not require special treatment and may be a closer mechanical analog to the human foot. Finally, though we treated the foot as a rigid object the ground forces of the prediction OCP are relatively smooth due to the inclusion of the impulses in the cost function. Though the inclusion of the impulse term improved our simulation results, the experimental work of Hoyt and Taylor (1981) and later Farley and Taylor (1991) suggests that terms for both muscular effort and ground contact terms should appear in cost functions used to predict legged locomotion.

## Data Availability

All datasets analyzed for this study are included in the manuscript and the Supplementary Files.

## Ethics Statement

The recordings were conducted according to the guidelines of the Declaration of Helsinki 2013 and approved by the ethics committee of Faculteit der Gedrags-en Bewegingswetenschappen (Faculty of Behavioral and Movement Sciences) at Vrije Universiteit.

## Author Contributions

MM worked with KM to develop the proposal that funded this work. MM undertook the work and the writing. KM provided critical review during the preparation of the manuscript.

### Conflict of Interest Statement

The authors declare that the research was conducted in the absence of any commercial or financial relationships that could be construed as a potential conflict of interest.

## Footnotes

^1^https://github.com/ORB-HD/rbdl-orb

## Supplementary Material

Please see the supplemental data for videos, data, and code. The data is accompanied by scripts that can be used to generate the tables and plots that appear in the results section. In addition, the scripts can also generate plots that show activation profiles, constraint error profiles, Baumgarte stabilization forces, and the phase of each OCP.
