# Supplementary material for: A Quick Turn of Foot: Rigid Foot-Ground Contact Models for Human Motion Prediction
Source: Front Neurorobot. 2019 Aug 7;13:62. doi: 10.3389/fnbot.2019.00062 (PMC6693511; doi:10.3389/fnbot.2019.00062)
Supplement: Supplementary file 1 [file Data_Sheet_1.ZIP › SupplementaryMaterial/resultsData/figures/fig_tracking_sim.pdf]

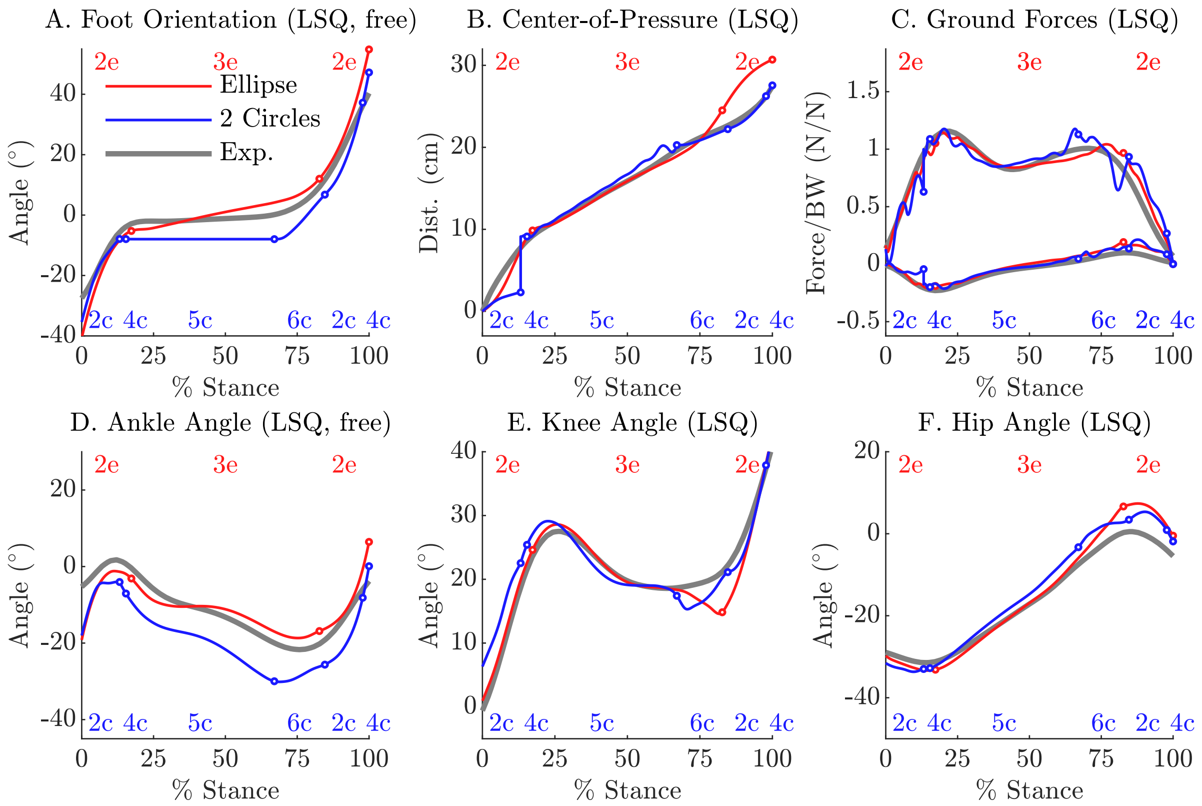
A. Foot Orientation (LSQ, free)
B. Center-of-Pressure (LSQ)
C. Ground Forces (LSQ)
D. Ankle Angle (LSQ, free)
E. Knee Angle (LSQ)
F. Hip Angle (LSQ)
Ellipse
2 Circles
Exp.
Angle (°)
Dist. (cm)
Force/BW (N/N)
% Stance
2e 3e 2e
2c 4c 5c 6c 2c 4c
