# Supplementary figures and images for: A Quick Turn of Foot: Rigid Foot-Ground Contact Models for Human Motion Prediction
Source: Front Neurorobot. 2019 Aug 7;13:62. doi: 10.3389/fnbot.2019.00062 (PMC6693511; doi:10.3389/fnbot.2019.00062)

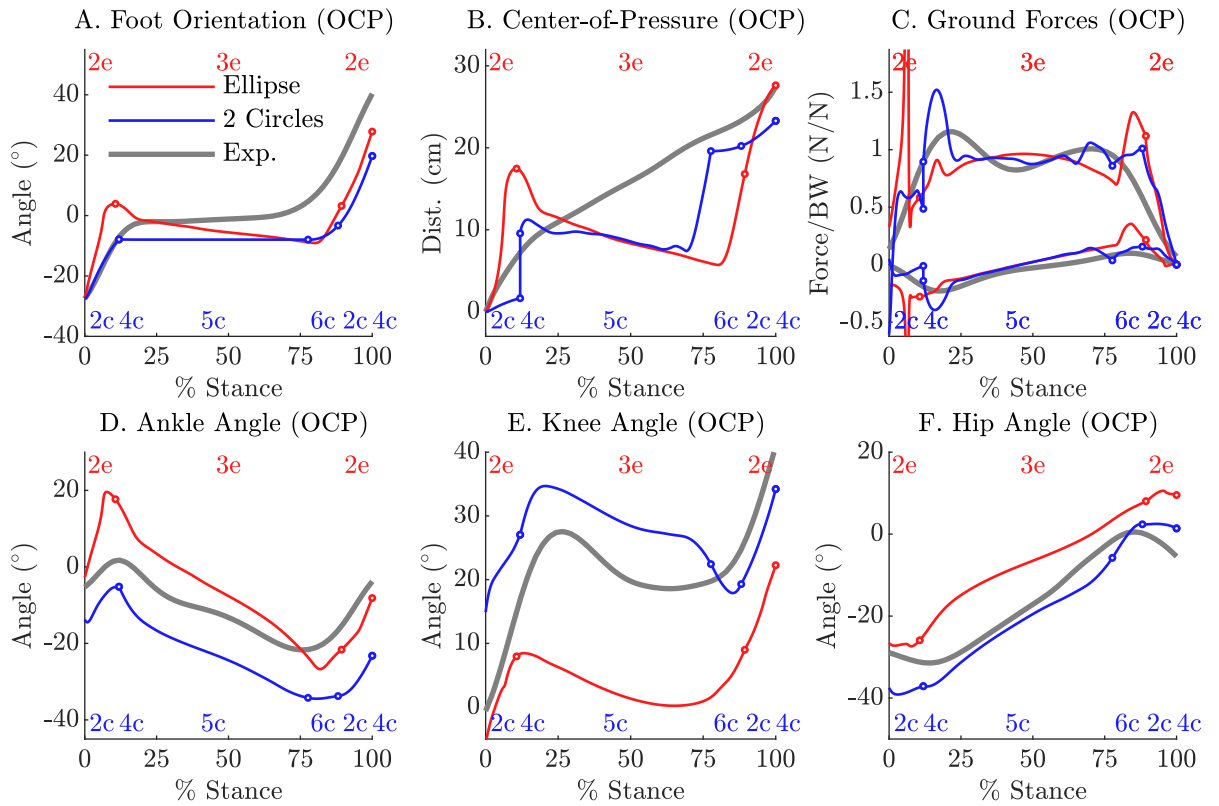

Supplement: Supplementary file 1 [file Data_Sheet_1.ZIP › SupplementaryMaterial/resultsData/figures/fig_prediction_sim.pdf]

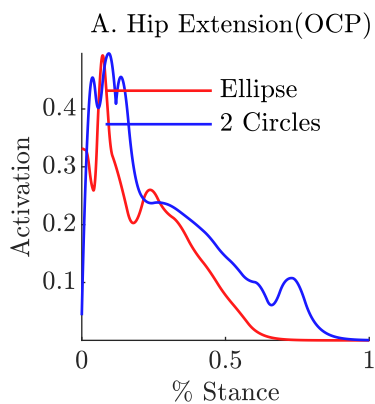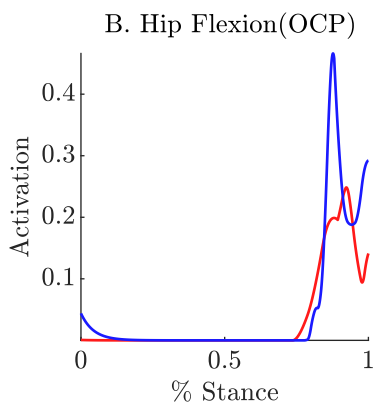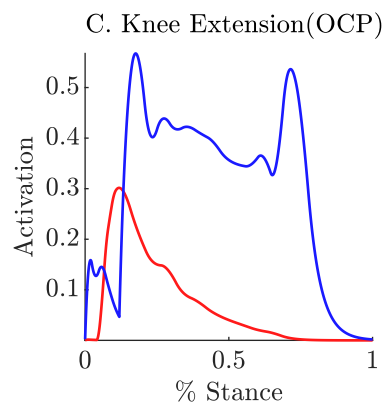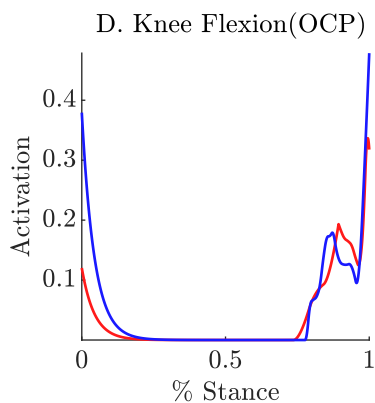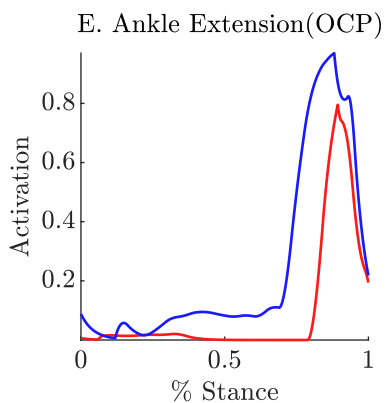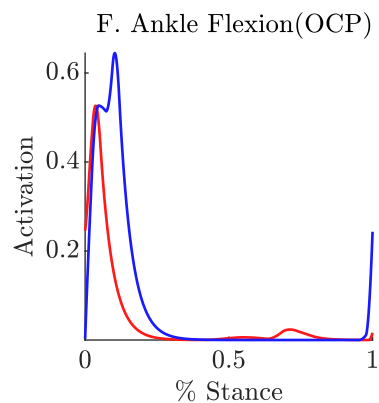

Supplement: Supplementary file 1 [file Data_Sheet_1.ZIP › SupplementaryMaterial/resultsData/figures/fig_prediction_stanceAct.pdf]

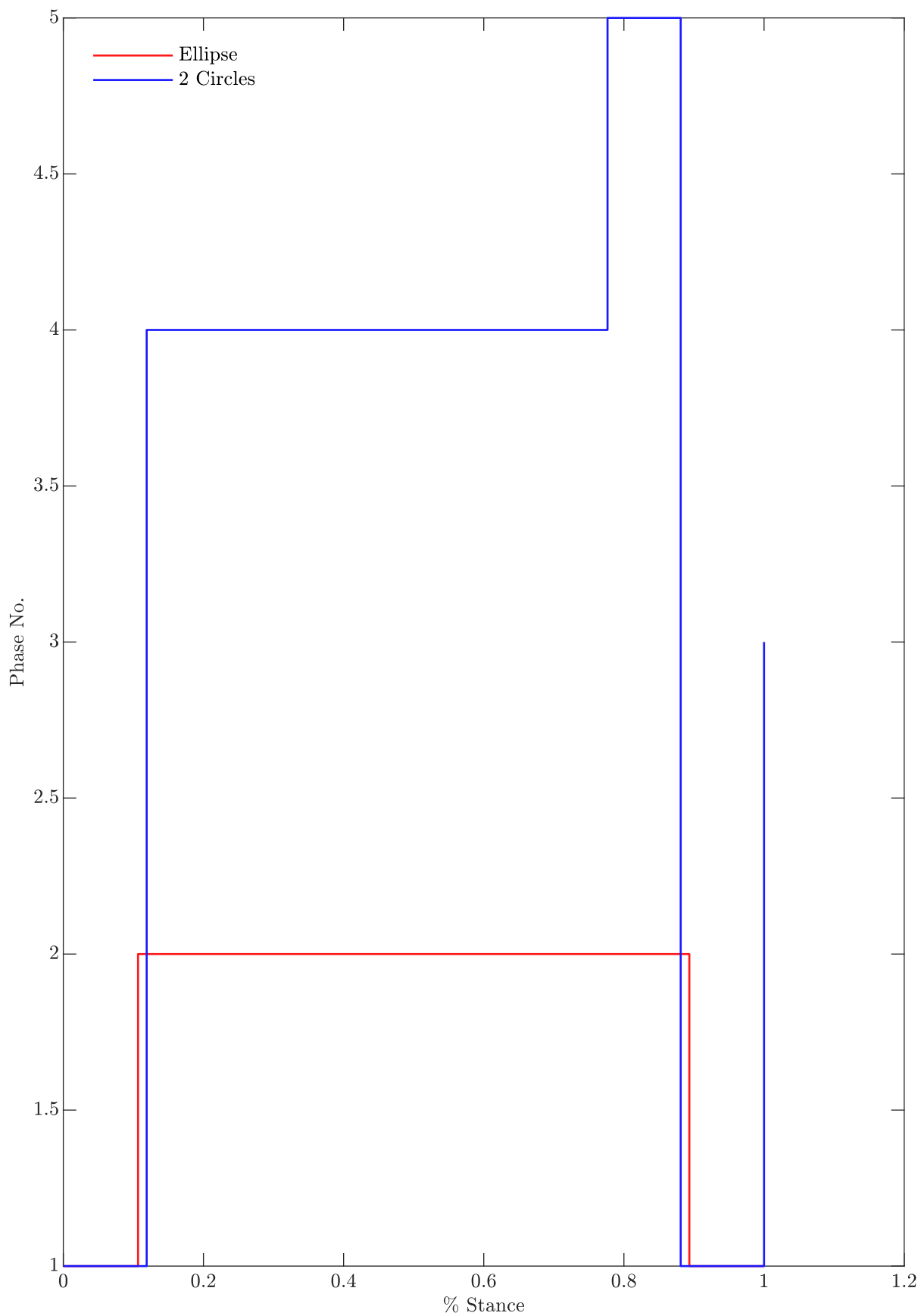

Supplement: Supplementary file 1 [file Data_Sheet_1.ZIP › SupplementaryMaterial/resultsData/figures/fig_prediction_stancePhases.pdf]

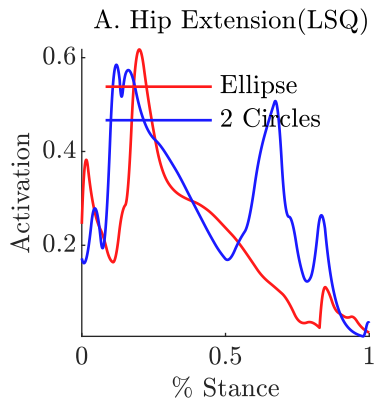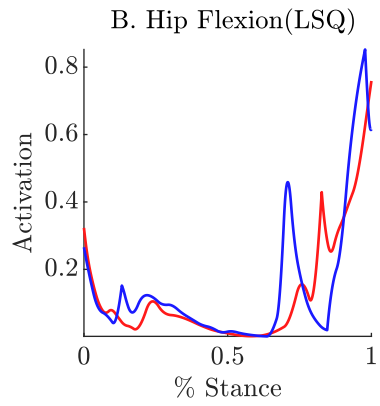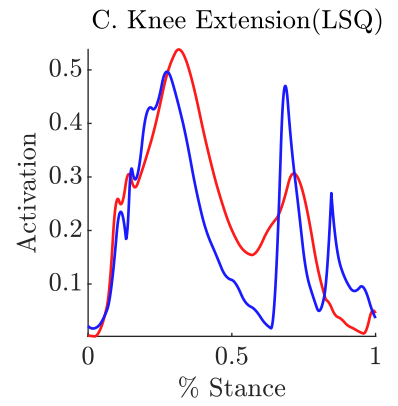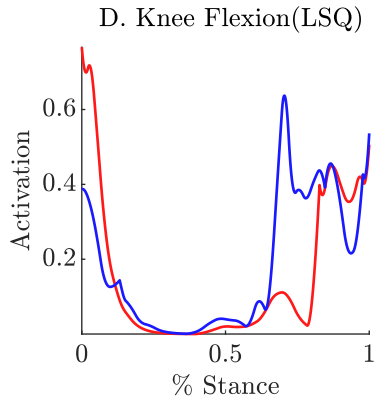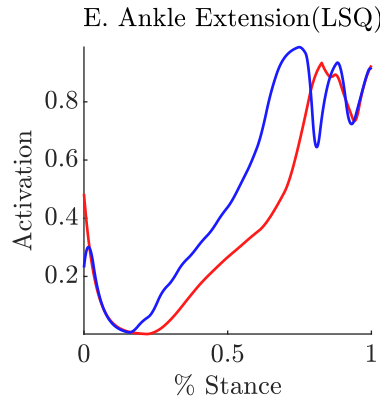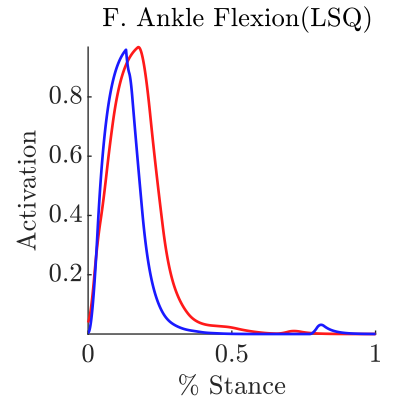

Supplement: Supplementary file 1 [file Data_Sheet_1.ZIP › SupplementaryMaterial/resultsData/figures/fig_tracking_stanceAct.pdf]

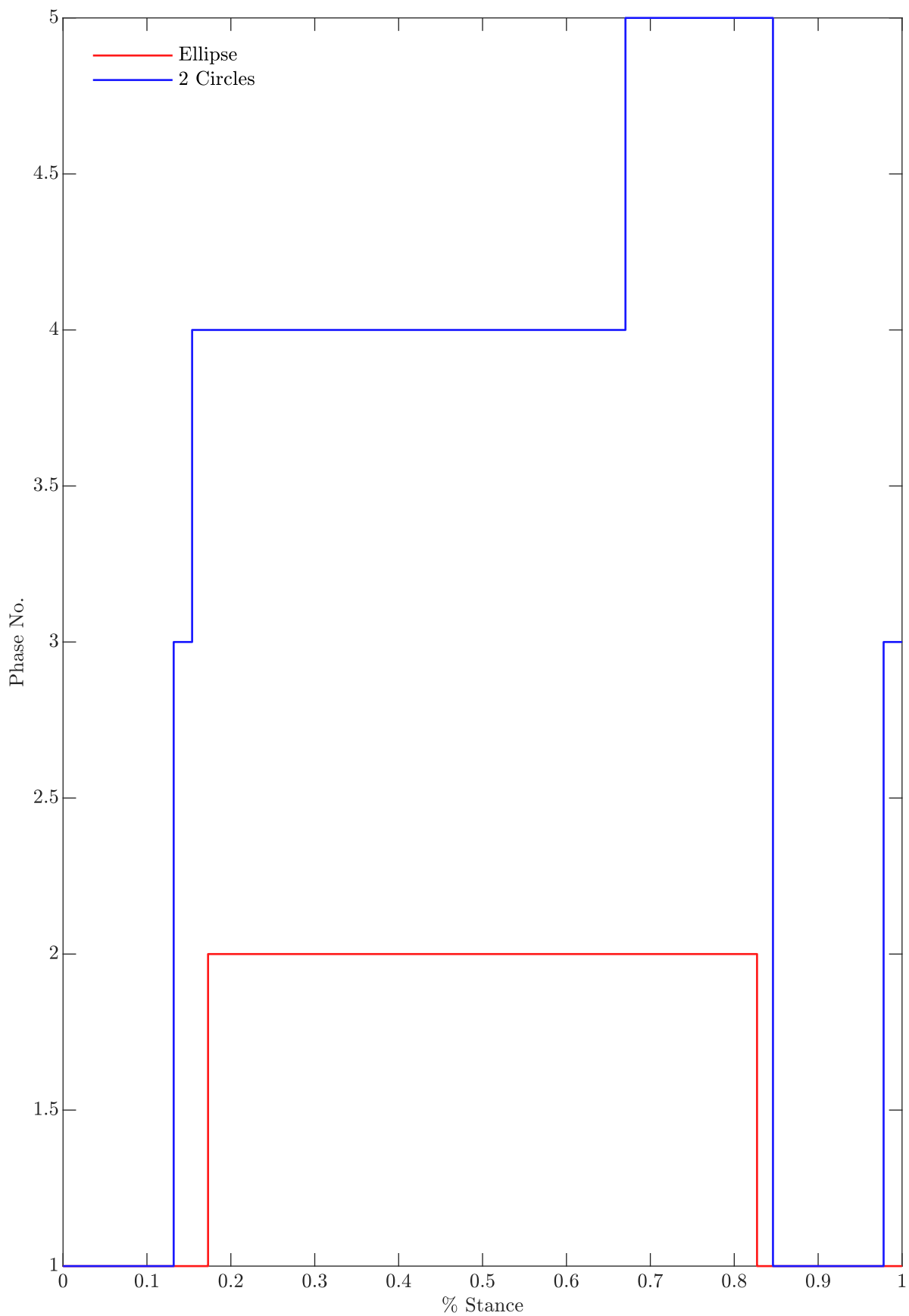

Supplement: Supplementary file 1 [file Data_Sheet_1.ZIP › SupplementaryMaterial/resultsData/figures/fig_tracking_stancePhases.pdf]
